# Proteomics for studying the effects of *L. rhamnosus* LV108 against non-alcoholic fatty liver disease in rats†

**DOI:** 10.1039/c8ra06771f

**Published:** 2018-11-15

**Authors:** Hengxian Qu, Hongbo Yu, Ruixia Gu, Dawei Chen, Xia Chen, Yingping Huang, Wenbo Xi, Yujun Huang

**Affiliations:** College of Food Science and Technology, Yangzhou University Yangzhou Jiangsu 225127 China; Key Lab of Dairy Biotechnology and Safety Control Yangzhou Jiangsu 225127 China; Uni-enterprise (China) Holding, Ltd. Kunshan Jiangsu 215300 China

## Abstract

Probiotics show protective effects against non-alcoholic fatty liver disease (NAFLD). However, their efficacy against NAFLD and the mechanisms are still unknown. In this study, Tandem Mass Tag (TMT) relative quantitative proteomics was utilized to track the changes in liver protein expression in rats fed with *Lactobacillus rhamnosus* LV108. A total of 4155 corresponding proteins were identified by MS. A total of 26 differentially expressed proteins were found between the *L. rhamnosus* LV108 treatment group and mode group, and there are 16 proteins up-regulated and 10 proteins down-regulated. Most of the differentially expressed proteins were involved in apoptosis and lipid metabolism. The key differentially expressed proteins (BFAR and Cyt-C) were verified by parallel reaction monitoring to be reliable. Our study is the first attempt to analyze the protein profile of probiotic-treated NAFLD model rats by quantitative proteomics. The identified proteins in this study will likely contribute to a better understanding of the molecular mechanisms of the effect of probiotics on NAFLD.

## Abstract 2

Probiotics show protective effects against non-alcoholic fatty liver disease (NAFLD).

## Graphical abstract

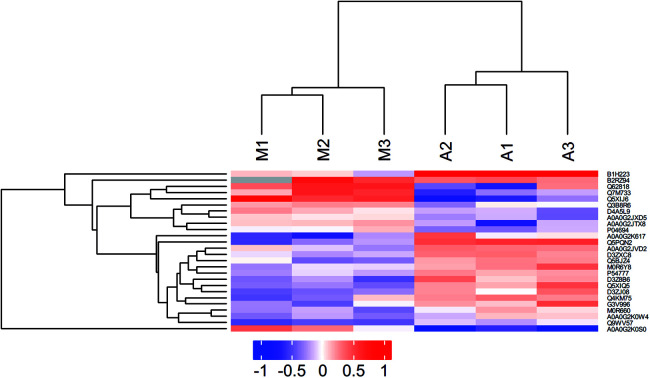

## Introduction

Non-alcoholic fatty liver disease (NAFLD) is one of the most common chronic liver diseases. Starting from the benign condition of simple steatosis, NAFLD may evolve in nonalcoholic steatohepatitis (NASH) and may progress to hepatic fibrosis, cirrhosis, and hepatocellular carcinoma.^1–3^ NAFLD is considered to be the hepatic manifestation of metabolic syndrome, as it is directly associated with insulin resistance (IR), central obesity, reduced glucose tolerance, type 2 diabetes mellitus (T2DM), arterial hypertension, and hypertriglyceridemia.^4^ The specific mechanism of NAFLD has not been fully elucidated but the “two-hit” theory^2^ has been widely accepted.^5^

Probiotics are known as the live microorganisms that can improve the intestinal flora and confer beneficial health effects on the host.^6,7^ A body of evidence suggests that probiotics have been discussed as hopeful alternatives in the treatment of NAFLD.^8^ Several strains of probiotics were shown to exhibit protective effects on NAFLD. In a high-fat diet model, *L. rhamnosus* and *acidophilus* mildly decreased intrahepatic lymphocytes and TNF-α expression.^9^*L. rhamnosus* GG impaired genes involved in hepatic inflammation and lipid metabolism in an NAFLD model induced by a high-fructose diet.^10^ Other investigations have reported that VSL#3 treatment reversed high-fat diet-induced depletion of hepatic natural killer T cells, as a consequence of direct decrease in pro-inflammatory cytokines, especially TNF-α and IκB.^11^ VSL#3 also reduced expression of peroxisome proliferator-activated receptor-α.^12^

Unlike the conventional molecular biological technologies, proteomics technologies enable investigators to define the global profile of protein expression in specific physical or pathological statuses.^13^ Most proteomic analyses of probiotic focus on the properties of probiotic^14^ and the molecular mechanisms of probiotic action.^15^ Proteomics has been used to explore the new markers of NAFLD^16^ and study the effects of some drugs or functional ingredients^17,18^ on NAFLD. Previous studies of the effect of probiotics on NAFLD have mainly focused on changes of some specific proteins or genes in specific pathways.^9,11,12^ The present research first adopted the method of proteomics to carry out differential analysis of liver proteome on NAFLD model rats treated with probiotic. Tandem Mass Tag (TMT) relative quantitative proteomics was utilized in this research, which displays changes in liver protein expression in rats among normal group (N), model group (M), and *L. rhamnosus* LV108 treatment group A (A). Furthermore, two differentially expressed proteins were selected for parallel reaction monitoring (PRM) analysis to further verify the results of MS. In addition, bioinformatics analysis was carried out to screen out the differential proteins which played a key role in the treatment of NAFLD by probiotic and provide valuable clues for deepening our understanding of its mechanism at the molecular level.

## Materials and methods

### Probiotic bacteria

*L. rhamnosus* LV108 (LV108) were provided by Jiangsu Key Lab of Dairy Biological Technology and Safety Control, China.

### Animals and treatment

Twenty-seven healthy male Sprague-Dawley rats were purchased from Comparative Medical Center of Yangzhou University, Jiangsu, China. The rats were 5 weeks old and weighing about 150 g at the start of the experiment. The animal experiments conformed the U.S. National Institutes of Health guidelines for the care and use of laboratory animals (NIH Publication no. 85-23 Rev. 1985) and were approved by the Animal Care Committee of the Center for Disease Control and Prevention (Jiangsu, China). All animals were housed under a 12 hour light/12 hour dark cycle in a controlled room with a temperature of 23 ± 3 °C and a humidity of 50% ± 10%. The animals were acclimated to their new circumstances for one week. Then rats were randomly divided into 3 groups (*n* = 9): normal group (N), model group (M), and *L. rhamnosus* LV108 treatment group A (A). Normal group fed a low fat diet (LFD: flour 20%, rice flour 10%, corn 20%, drum skin 26%, soy material 20%, fish meal 2%, bone meal 2%), and model group and *L. rhamnosus* LV108 treatment group A fed a high fat diet (HFD: 10% lard, 10% egg powder, 1% cholesterol and 0.2% bile salts and 78.8% LFD) for 8 weeks. All rats were allowed free access to food and water. From the fourth week all rats received the following treatments by lavage: normal group and model group rats: physiological saline (1 ml/100 g); *L. rhamnosus* LV108 treatment group A: *L. rhamnosus* LV108 fermented milk (1 ml/100 g, 10^9^ CFU ml^−1^). After 8 weeks, rats underwent 12 h of fasting prior to being anaesthetized and dissected. All rats were euthanized at the anestrus period following anesthesia under 1% sodium pentobarbital. Livers were removed and stored at −80 °C for subsequent analyses.

### Protein extraction and normalization

SDT buffer was added to the liver sample, and transferred to 2 ml tubes with amount quartz sand (another 1/4 inch ceramic bead MP 6540-424 for tissue samples). The lysate was homogenized by MP homogenizer (24 × 2, 6.0M/S, 60 s, twice). The homogenate was sonicated and then boiled for 15 min. After centrifuged at 14 000 g for 40 min, the supernatant was filtered with 0.22 μm filters. The filtrate was quantified with the BCA Protein Assay Kit (Bio-Rad, USA). The sample was stored at −80 °C. Equivalent amounts of protein from each of three different rats were pooled to generate three protein samples for each group.

### SDS-PAGE separation

20 μg of proteins for each sample were mixed with 5× loading buffer respectively and boiled for 5 min. The proteins were separated on 12.5% SDS-PAGE gel (constant current 14 mA, 90 min). Protein bands were visualized by Coomassie Blue R-250 staining.

### FASP digestion

200 μg of proteins for each sample were incorporated into 30 μl SDT buffer (4% SDS, 100 mM DTT, 150 mM Tris–HCl pH 8.0). The detergent, DTT and other low-molecular-weight components were removed using UA buffer (8 M urea, 150 mM Tris–HCl pH 8.0) by repeated ultrafiltration (Microcon units, 10 kD). Then 100 μl iodoacetamide (100 mM IAA in UA buffer) was added to block reduced cysteine residues and the samples were incubated for 30 min in darkness. The filters were washed with 100 μl UA buffer three times and then 100 μl 100 mM TEAB buffer twice. Finally, the protein suspensions were digested with 4 μg trypsin (Promega) in 40 μl TEAB buffer overnight at 37 °C, and the resulting peptides were collected as a filtrate. The peptide content was estimated by UV light spectral density at 280 nm using an extinctions coefficient of 1.1 of 0.1% (g l^−1^) solution that was calculated on the basis of the frequency of tryptophan and tyrosine in vertebrate proteins.

### TMT labeling

Peptide mixture of each sample (100 g) was labeled using TMT reagent according to the manufacturer's instructions (Thermo Fisher Scientific).

### Peptide fractionation with high pH reversed-phase

Pierce high pH reversed-phase fractionation kit (Thermo scientific) was used to fractionate TMT-labeled digest samples into 10 fractions by an increasing acetonitrile step-gradient elution according to instructions.

### Mass spectrometry

#### HPLC

Each fraction was injected for nanoLC-MS/MS analysis. The peptide mixture was loaded onto a reverse phase trap column (Thermo Scientific Acclaim PepMap100, 100 μm × 2 cm, nanoViper C18) connected to the C18-reversed phase analytical column (Thermo Scientific Easy Column, 10 cm long, 75 μm inner diameter, 3 μm resin) in buffer A (0.1% formic acid) and separated with a linear gradient of buffer B (84% acetonitrile and 0.1% formic acid) at a flow rate of 300 nl min^−1^ controlled by IntelliFlow technology.

1.5 hours gradient: 0–55% buffer B for 80 min, 55–100% buffer B for 5 min, hold in 100% buffer B for 5 min.

#### LC-MS/MS analysis

LC-MS/MS analysis was performed on a Q Exactive mass spectrometer (Thermo Scientific) that was coupled to Easy nLC (Proxeon Biosystems, now Thermo Fisher Scientific) for 60 min (determined by project proposal). The mass spectrometer was operated in positive ion mode. MS data was acquired using a data-dependent top10 method dynamically choosing the most abundant precursor ions from the survey scan (300–1800 *m*/*z*) for HCD fragmentation. Automatic gain control (AGC) target was set to 3 × 10^6^, and maximum inject time to 10 ms. Dynamic exclusion duration was 40.0 s. Survey scans were acquired at a resolution of 70 000 at *m*/*z* 200 and resolution for HCD spectra was set to 35 000 at *m*/*z* 200, and isolation width was 2 *m*/*z*. Normalized collision energy was 30 eV and the underfill ratio, which specifies the minimum percentage of the target value likely to be reached at maximum fill time, was defined as 0.1%. The instrument was run with peptide recognition mode enabled.

### Data analysis

MS/MS spectra were searched using MASCOT engine (Matrix Science, London, UK; version 2.2) embedded into Proteome Discoverer 1.4.

### Bioinformatics analysis

#### Gene ontology (GO) functional annotation

The protein sequences of differentially expressed proteins were in batches retrieved from UniProtKB database (Release 2016_10) in FASTA format. The retrieved sequences were locally searched against SwissProt database (mouse) using the NCBI BLAST + client software (ncbi-blast-2.2.28+-win32.exe) to find homologue sequences, from which the functional annotation can be transferred to the studied sequences. In this work, the top 10 blast hits with *E*-value less than 1 × 10^−3^ for each query sequence were retrieved and loaded into Blast2GO (Version 3.3.5) for GO mapping and annotation. In this work, an annotation configuration with an *E*-value filter of 1 × 10^−6^, default gradual EC weights, a GO weight of 5, and an annotation cutoff of 75 were chosen. Un-annotated sequences were then re-annotated with more permissive parameters. The sequences without BLAST hits and un-annotated sequences were then selected to go through an InterProScan against EBI databases to retrieve functional annotations of protein motifs and merge the InterProScan GO terms to the annotation set. The GO annotation results were plotted by R scripts.

#### KEGG pathway annotation

The FASTA protein sequences of differentially changed proteins were blasted against the online Kyoto Encyclopedia of Genes and Genomes (KEGG) database to retrieve their KOs and were subsequently mapped to pathways in KEGG. The corresponding KEGG pathways were extracted.

#### Functional enrichment analysis

To further explore the impact of differentially expressed protein in cell physiological process and discover internal relations between differentially expressed proteins, enrichment analysis was performed. GO enrichment on three ontologies (biological process, molecular function, and cellular component) and KEGG pathway enrichment analyses were applied based on the Fisher’ exact test, considering the whole quantified protein annotations as background dataset. Benjamini–Hochberg correction for multiple testing was further applied to adjust derived *p*-values. Only functional categories and pathways with *p*-values under a threshold of 0.05 were considered as significant.

#### Hierarchical clustering

The protein relative expression data was selected for hierarchical clustering analysis, and Cluster3.0 and the Java Treeview software were used. Euclidean distance algorithm for similarity measure and average linkage clustering algorithm (clustering uses the centroids of the observations) for clustering were selected when performing hierarchical clustering. Heatmap is often presented as a visual aid in addition to the dendrogram.

### PRM analysis

Liquid chromatography-coupled targeted mass spectrometry analysis was performed by injecting the column with 2 μg of peptide, with 20 fmol of each SIS peptide spiked in. Peptides were separated using the Easy-nLC 1200 (Thermo Scientific). Mass spectrometry analysis was performed using the PRM mode on a Q-Exactive HF mass spectrometer (Thermo Scientific). PRM data analysis was performed using Skyline 3.5.0 software.

## Results

### LC-MS/MS analysis

The results indicated that each group had a high reproducibly (*R* > 0.5) between the repeats (ESI Enclosure 1†). A total of 4155 corresponding proteins were identified by MS. Proteins that conformed to the following screening criteria were deemed as differentially expressed: 1.2-fold for up-regulated proteins and of 0.83-fold for down-regulated proteins (*P* < 0.05). A total of 198 differentially expressed proteins (103 increased, 95 decreased) were found between M group and N group (ESI Table S1†). A total of 26 differentially expressed proteins (16 increased, 10 decreased) were found between A group and M group (Table 1).

**Table 1:** Differentially expressed proteins between groups

Accession	Protein	A/M	*P* value
Q5PQN2*	Bifunctional apoptosis regulator	1.717 951 603	0.000 495 094
D3ZXC8	Emopamil binding protein-like	1.296 521 452	0.001 231 869
P54777	Peroxisome assembly factor 2	1.24 280 249	0.002 351 283
Q9WV57	Macrophage-expressed gene 1 protein	1.2 603 632	0.002 533 069
B1H223	Down syndrome critical region gene 3	1.843 374 235	0.00306368
Q5XIQ5	Protein SDA1 homolog	1.452 839 873	0.007 849 402
D3Z8B6	Abhydrolase domain-containing 15	1.44 066 251	0.010 973 197
D3ZJ08	Histone H3	1.411 730 878	0.012 718 204
A0A0G2JVD2	Solute carrier family 25 member 23	1.261 436 297	0.01 430 625
Q5BJZ4	Surfeit 6	1.318 850 738	0.018 714 614
M0R660	Glyceraldehyde-3-phosphate dehydrogenase	1.237 223 397	0.023 972 182
M0R6Y8	Phosphoglycerate kinase	1.302 525 319	0.024 559 267
Q4KM75	CD5 antigen-like	1.354 239 507	0.037 254 603
A0A0G2K0W4*	Leukocyte receptor cluster member 8	1.208 548 489	0.039 674 725
A0A0G2K617	RWD domain-containing protein	1.489 288 396	0.048 550 643
G3V996	LETM1 domain-containing 1	1.372 158 051	0.049 273 179
Q5XIJ6*	BRISC and BRCA1-A complex member 1	0.467 797 475	0.00 241 747
A0A0G2K0S0	Phosphatase and actin regulator	0.43 316 719	0.00 950 649
A0A0G2JXD5	Beta-chimaerin	0.821 693 632	0.011 748 677
Q7M733	Hermansky–Pudlak syndrome 6 protein homolog	0.608 667 807	0.019 329 632
D4A5L9	Similar to cytochrome *c*, somatic	0.798 758 983	0.021 463 428
P04694	Tyrosine aminotransferase	0.83 150 074	0.024 512 388
Q3B8R6*	Alpha-2-glycoprotein 1, zinc	0.829 655 449	0.034 740 363
Q62818*	Translation initiation factor eIF-2B subunit beta	0.614 987 199	0.037 041 167
A0A0G2JTX8	CAP-Gly domain-containing linker protein 1	0.754 347 039	0.04 291 738
B2RZ94*	RGD1563239 protein	0.821 109 353	0.047 760 262

The proteins, which were differentially expressed in A/M and M/N and showed differences in the opposite variation trend, were further screened. A total of 6 optimized differentially expressed proteins were identified. Among them, Translation initiation factor eIF-2B subunit beta, BRISC and BRCA1-A complex member 1, RGD1563239 protein and Alpha-2-glycoprotein 1, zinc were up-regulated in M group but could be corrected after probiotic treatment. Bifunctional apoptosis regulator and Leukocyte receptor cluster member 8 were down-regulated in M group but could be corrected after probiotic treatment.

### Hierarchical clustering analysis

**Fig. 1:**
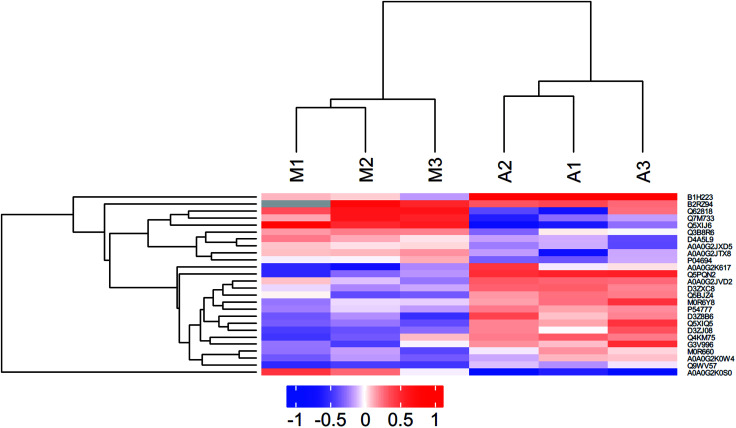
Hierarchical clustering of changes in abundance of the differentially expressed proteins. Hierarchical clustering analysis. Through horizontal comparison, samples could be classified into three categories, suggesting that the selected differentially expressed proteins could effectively distinguish samples. Vertical comparison indicated that proteins could be classified into two categories with opposite directional variation, demonstrating the rationality of the selected differentially expressed proteins.

Hierarchical clustering results were expressed as a tree heat map (Fig. 1). X-coordinates represented sample and Y-coordinates differentially expressed proteins. log2-expression of differentially expressed proteins in tested samples was displayed in different colors in the heat map, with red representing up-regulation and green indicating down-regulation. The differentially expressed proteins screened in this study can effectively separate the M from the A. The hierarchical clustering analysis thus supported that the differentially expressed proteins screened out were reasonable.

### GO functional annotation analysis

In proteomics research, the object of the study is a collection of all proteins in a cell, tissue and organism. For high-throughput omics, understanding which functions or biological pathways are significantly affected by biological processing is a top priority. Therefore, the proteins and their functions need to be summarized and analyzed from a more systematic level. Using the Blast2Go software, GO functional annotation on all proteins was identified in this study. Then, the differentially expressed proteins were subjected to GO enrichment analysis by Fisher's exact test.

**Fig. 2:**
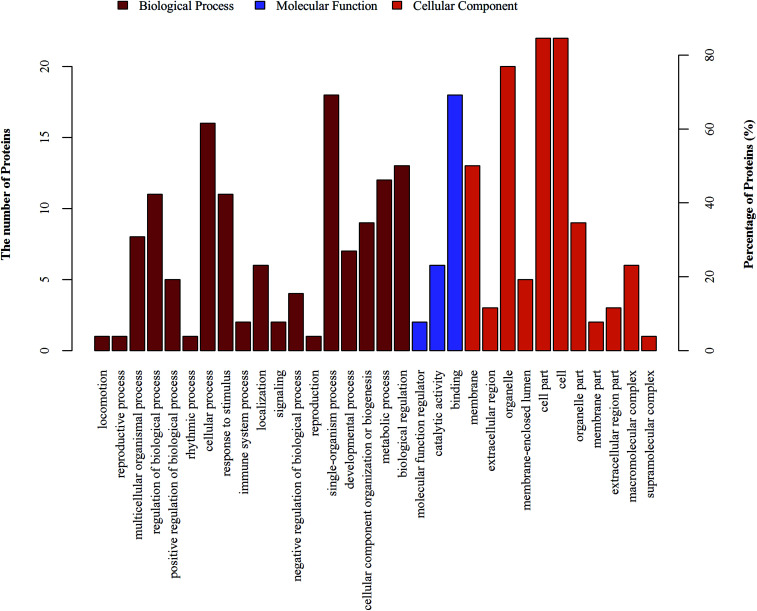
GO functional annotation analysis of the differentially expressed proteins. GO functional annotation analysis. The differentially expressed proteins are mainly annotated as binding, cell part, and single-organism process in terms of molecular function, cell composition, and biological process, respectively.

The 26 differentially expressed proteins were categorized into biological processes, cellular components, and molecular functions according to their annotation (Fig. 2). The most prevalent biological processes were single-organism process (18), cellular process (16), single-organism cellular process (15), and biological regulation (13). The most prevalent cellular components were located in the cell part (22), cell (22) and intracellular (22). The most predominant molecular functions were binding (18) and protein binding (14).

**Fig. 3:**
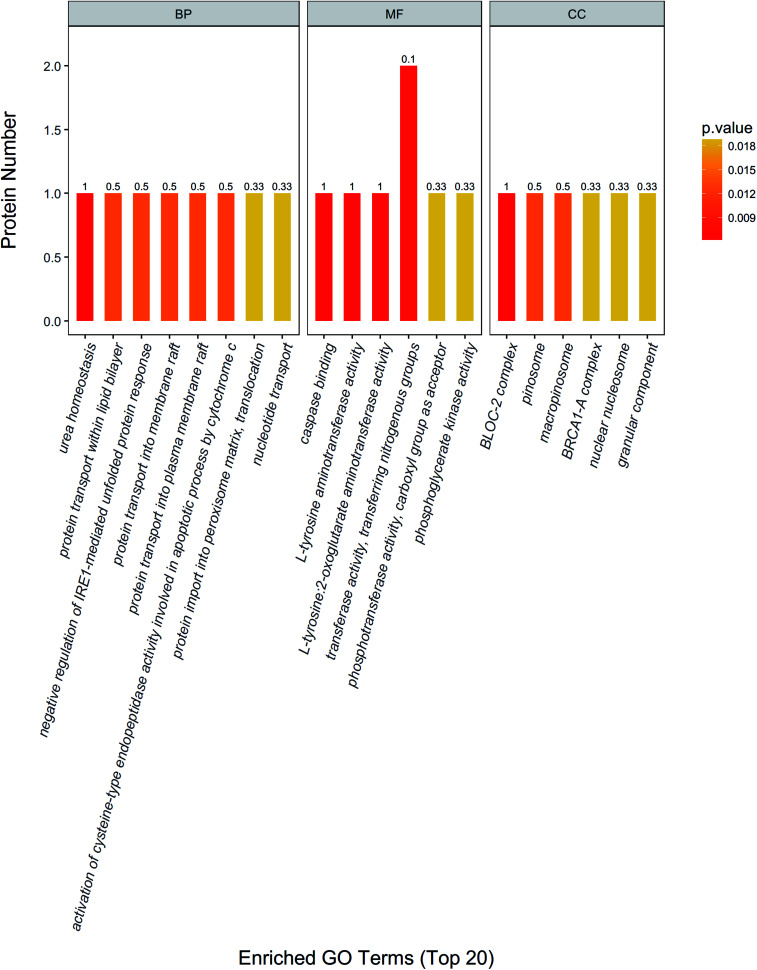
GO functional enrichment analysis of the differentially expressed proteins. GO functional enrichment analysis. Urea homeostasis and other important biological processes change significantly. Caspase binding and other molecular function change significantly. BLOC-2 complex and other positioning proteins change significantly.

In addition, urea homeostasis, protein transport within lipid bilayer, negative regulation of IRE1-mediated unfolded protein response, protein transport into membrane raft, activation of cysteine-type endopeptidase activity involved in apoptotic process by cytochrome *c*, protein import into peroxisome matrix, translocation, nucleotide transport and other important biological processes change significantly. Caspase binding, l-tyrosine aminotransferase activity, l-tyrosine: 2-oxoglutarate aminotransferase activity, transferase activity, transferring nitrogenous groups, phosphotransferase activity, carboxyl group as acceptor, phosphoglycerate kinase activity and other molecular function change significantly. BLOC-2 complex, pinosome, macropinosome, BRCA1-A complex, nuclear nucleosome, granular component and other positioning proteins change significantly (Fig. 3).

### KEGG pathway analysis

**Fig. 4:**
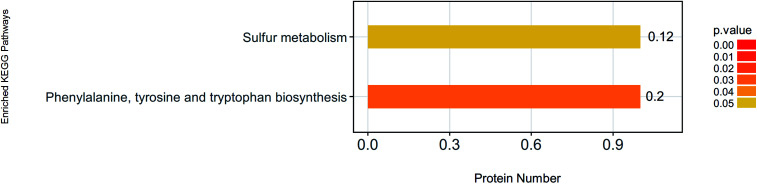
KEGG pathway analysis of the significantly changed pathways. KEGG pathway analysis. Phenylalanine, tyrosine and tryptophan biosynthesis, and sulfur metabolism pathways had changed significantly. TAT and Cyt-C were identified as the important signal molecules of these pathways.

Protein cannot exercise of their functions independently. Instead, different proteins coordinate with each other to complete a series of biochemical reactions to exercise their biological functions. Using KEGG pathway analysis, we enriched 37 KEGG pathways (Table 2). Phenylalanine, tyrosine and tryptophan biosynthesis, and Sulfur metabolism pathways had changed significantly (Fig. 4). P04694 (tyrosine aminotransferase) and D4A5L9 (cytochrome *c*, Cyt-C) were identified as the important signal molecules of these pathways. We also found that Cyt-C was involved in multiple pathways such as influenza A, hepatitis B, viral myocarditis, non-alcoholic fatty liver disease (NAFLD), platinum drug resistance, tuberculosis, parkinson's disease, colorectal cancer, apoptosis-multiple species, toxoplasmosis, herpes simplex infection, pathways in cancer, amyotrophic lateral sclerosis (ALS), apoptosis, kaposi's sarcoma-associated herpesvirus infection, p53 signaling pathway, small cell lung cancer and huntington's disease (Table 2). In these pathways, Cyt-C was mainly involved in the process of apoptosis.

**Table 2:** KEGG pathway analysis of the differentially expressed proteins

Map ID	Map name	Seqs	Seqs num
map05010	Alzheimer's disease	M0R660 D4A5L9	2
map01230	Biosynthesis of amino acids	M0R660 P04694	2
map04146	Peroxisome	P54777	1
map05164	Influenza A	D4A5L9	1
map00010	Glycolysis/gluconeogenesis	M0R660	1
map05161	Hepatitis B	D4A5L9	1
map04150	mTOR signaling pathway	A0A0G2JTX8	1
map05416	Viral myocarditis	D4A5L9	1
map04932	Non-alcoholic fatty liver disease (NAFLD)	D4A5L9	1
map01524	Platinum drug resistance	D4A5L9	1
map00920	Sulfur metabolism	D4A5L9	1
map05152	Tuberculosis	D4A5L9	1
map00360	Phenylalanine metabolism	P04694	1
map05012	Parkinson's disease	D4A5L9	1
map01200	Carbon metabolism	M0R660	1
map05322	Systemic lupus erythematosus	D3ZJ08	1
map05134	Legionellosis	D4A5L9	1
map00400	Phenylalanine, tyrosine and tryptophan biosynthesis	P04694	1
map05210	Colorectal cancer	D4A5L9	1
map04215	Apoptosis – multiple species	D4A5L9	1
map05145	Toxoplasmosis	D4A5L9	1
map05168	Herpes simplex infection	D4A5L9	1
map03440	Homologous recombination	Q5XIJ6	1
map05200	Pathways in cancer	D4A5L9	1
map03013	RNA transport	Q62818	1
map00270	Cysteine and methionine metabolism	P04694	1
map05034	Alcoholism	D3ZJ08	1
map05203	Viral carcinogenesis	D3ZJ08	1
map05014	Amyotrophic lateral sclerosis (ALS)	D4A5L9	1
map04210	Apoptosis	D4A5L9	1
map05167	Kaposi's sarcoma-associated herpesvirus infection	D4A5L9	1
map04115	p53 signaling pathway	D4A5L9	1
map00130	Ubiquinone and other terpenoid-quinone biosynthesis	P04694	1
map00350	Tyrosine metabolism	P04694	1
map04066	HIF-1 signaling pathway	M0R660	1
map05222	Small cell lung cancer	D4A5L9	1
map05016	Huntington's disease	D4A5L9	1

### PRM analysis

To further verify the results of MS, 2 differentially expressed proteins (D4A5L9: cytochrome *c*: and Q5PQN2: bifunctional apoptosis regulator) were selected for PRM analysis.

The Skyline analysis results of each of 5 target peptide fragments of the 2 target proteins are shown in ESI Enclosure 2,† including information such as chromatographic peaks of peptide fragments, original peak area, and comparison histogram of the original peak area. Three possibly continuous daughter ions with high peptide fragment abundance were selected for quantitative analysis. After Skyline analysis were exported, peak area results of each target peptide fragment are shown in ESI Table S2.† These include target protein name, target peptide fragment sequence, parent ion charge, daughter ion selected, charge, and original peak area for quantification. It can be observed that all 5 peptide fragments provided quantitative information in the 9 samples. However, some of these peptides have weak quantitative information in some samples. ESI Table S3† displays the detailed data analysis results including quantitative information at the peptide fragment level, data calibration, and biological statistical analysis. First, daughter ion peak areas of the target peptide fragments were integrated, to obtain the original peak area of the peptide fragment in each sample of the different groups. Subsequently, the peak area of the heavy isotope-labeled internal standard peptide fragment was used for adjustment, to obtain the relative expression quantities of each peptide fragment in the different samples, with the adjusted value being the ratio of the original peak area of the peptide fragment to that of the incorporated heavy isotope-labeled peptide fragment in the sample. Finally, the mean relative expression quantity of the target peptide fragment in each group of samples was calculated, and statistical analysis was carried out, which is shown in Table 3.

**Table 3:** Target peptide fragment PRM analysis

Peptide sequence	Protein name	*N*-mean	*M*-mean	*A*-mean	Ratio_*M*/*N*	Ratio_*A*/*N*	Ratio_*A*/*M*
TGPNLHGLFGR	D4A5L9	2.1352	2.2511	2.1047	1.0543	0.9857	0.9350
TGQAAGFSYTDANK	D4A5L9	3.8698	3.9754	2.5038	1.0273	0.6470	0.6298
ADLIAYLK	D4A5L9	1.4538	1.0469	0.5471	0.7201	0.3763	0.5226
LFPDAIK	Q5PQN2	0.1041	0.2012	0.1720	1.9323	1.6516	0.8547
VEDIQQNNDVVQSLAAFQK	Q5PQN2	0.0015	0.0015	0.0012	1.0568	0.8533	0.8074

Difference in the relative expression quantity of target proteins was further calculated based on that of the corresponding peptide fragment of each target protein among different sample groups (Table 4, ESI Table S3†).

**Table 4:** Target protein PRM analysis

Protein name	*N*-mean	*M*-mean	*A*-mean	Ratio_*M*/*N*	Ratio_*A*/*N*	Ratio_*A*/*M*
D4A5L9	2.4862	2.4245	1.7185	0.9751	0.6912	0.7088
Q5PQN2	0.0528	0.1014	0.0866	1.9203	1.6405	0.8543

The results of LC-PRM/MS analysis performed on 5 peptide fragments of 2 target proteins from 3 groups of liver samples showed that quantitative information of target peptide fragments could be obtained in all 9 samples. Relative quantitative analysis was carried out on target peptide fragments and proteins through the incorporation of heavy isotope-labeled peptide fragments.

The results indicated that the expression quantities of Q5PQN2 in the M group were markedly up-regulated compared with those in the N group, whereas the expression quantity of it in the A group was down- regulated compared with that in the M group. Furthermore, expression quantities of D4A5L9 in the A group were markedly down-regulated compared with that in the M group. The expression quantities of D4A5L9 in the M group were not significantly changed compared with that in the N group.

## Discussion

NAFLD is the most common chronic liver disease, comprises simple steatosis, steatohepatitis, fibrosis, cirrhosis, and hepatocellular carcinoma.^19^ The hallmark of NAFLD is the intracellular accumulation of lipids, resulting in the formation of lipid droplets within hepatocytes. This accumulation results from an imbalance between lipid synthesis and oxidation.^20–22^ We successfully established a NAFLD model by using a high-fat diet. To the best of our knowledge, it is the first proteomics report regarding the effect of probiotics on NAFLD. The obtained data have notably discovered relevant information of some new differentially expressed proteins, which is of considerable significance toward promoting research on the mechanism of probiotics on NAFLD.

Apoptosis is a programmed form of cell death that is considered to be a key component of various physiologic processes. Hepatocyte apoptosis is prevalent in all stages of the development of NAFLD.^23,24^ Bifunctional apoptosis regulator (BFAR) is a protein that inhibits the apoptotic signaling pathway and it was first discovered by Reed *et al.*^25^ Two major pathways for induction of apoptosis have been identified-intrinsic and extrinsic.^26–28^ BFAR is a protein at the intersection of two major pathways controlling apoptosis. The BFAR protein contains a SAM domain, which is required for its interactions with Bcl-2 and Bcl-X(L) and for suppression of Bax-induced cell death in both mammalian cells and yeast. In addition, BFAR contains a DED-like domain responsible for its interaction with DED-containing procaspases and suppression of Fas-induced apoptosis.^25^ It was observed in this study that BFAR expression level was down-regulated in M group, but it could be corrected after LV108 treatment. As far as we know, there are no reports indicate that probiotics can promote the expression of BFAR. However, some studies had shown that probiotics can regulate Fas/FasL, Bax/Bcl-2 and caspases.^29–31^ We believe that BFAR is an important target for LV108 to inhibit hepatocyte apoptosis and alleviate NAFLD.

KEGG pathway analysis shown that Sulfur metabolism pathways had changed significantly, with Cyt-C being identified as the important signal molecules of that pathway. Sulfur metabolism is an important metabolic process, and its metabolite, cysteine, regulates cell apoptosis. It involves the development of a variety of metabolic diseases such as atherosclerosis, obesity and diabetes.^32–35^ Cyt-C released by mitochondria is an important apoptosis-inducing factor. A second major pathway for apoptosis involves the participation of mitochondria, which releases cytochrome *c* (Cyt-C), resulting in caspase activation through the effects of apoptotic protease-activating factor-1(Apaf-1).^36,37^ Pre-treatment with probiotic significantly prevented release of Cyt-C to cytosol.^29^ In this study, it was observed that compared with the M group, Cyt-C expression level was down-regulated after LV108 treatment. This means that apoptosis was inhibited. We also found that Cyt-C was involved in multiple pathways such as NAFLD, hepatitis B, apoptosis-multiple species, apoptosis and p53 signaling pathway. It is a protein that was enriched in the most pathways of all differential proteins in the A/M group. In these pathways, Cyt-C is mainly involved in the process of apoptosis. In the NAFLD pathway, Cyt-C binds to Apaf-1 to activate caspase-9, and the activated caspase-9 activates caspase-3 and caspase-7. Eventually, it leads to apoptosis and hepatocellular injury. The results indicate that LV108 modulate crucial points of apoptosis. We hypothesize that LV108 attenuates NAFLD by inhibiting apoptosis and alleviating hepatocellular injury.

In this study, it was also observed that CD5 antigen-like (CD5L) expression level was up-regulated after LV108 treatment. It further illustrates that the LV108 has an inhibitory effect on apoptosis. CD5L, a soluble protein belonging to the SRCR superfamily, is expressed mostly by macrophages in lymphoid and inflamed tissues.^38^ CD5L has been suggested to exhibit different functions to various types of target cells.^39^ It is identified as a critical factor of protecting macrophages from the apoptotic effects of oxidized lipids.^40,41^ Moreover, the expression of CD5L is transcriptionally controlled by LXRs^42^ and plays major roles in lipid homeostasis.^43^ CD5L decreases the level of polyunsaturated fatty acyls (PUFA), affecting the expression of key cholesterol biosynthesis enzymes.^44^ In addition, CD5L is a target gene for SREBP-1a, a transcription factor that positively regulates lipogenic genes.^45^ The development and progression of NAFLD is characterized by hepatocellular redox imbalance, which may depend on the impaired regulation of lipid metabolism.^46^ When the expression level of CD5L was up-regulated, the biosynthesis of cholesterol was reduced and the lipid metabolism was restored to balance. LV108 alleviated lipid metabolism disorders by regulating key proteins that modulate the balance of lipid metabolism. It will be an important direction for our follow-up study.

CD5L has been placed at the intersection between lipid homeostasis and immune response. The same is true for α/β-hydrolase domain containing protein 15(ABHD15). ABHD15 is a type of lipoprotein and widely expressed, with highest expression in adipose tissue, liver, and skeletal muscle.^47^ ABHD15 is a direct and functional target gene of peroxisome proliferator-activated receptor gamma (PPARγ), the master regulator of adipogenesis.^48^ It is noteworthy that although the expression of ABHD15 was increased during adipogenesis, it was reduced in physiological situations with high free fatty acid levels like high-fat diet.^49^ High levels of FFAs might lead to decreased expression of ABHD15. On the other hand, Abhd15 knockdown results in increased apoptosis, whereas induction of apoptosis increases Abhd15 expression, suggesting a protective role of ABHD15 against apoptosis.^48^ In this study, it was observed that compared with the M group, the expression level of ABHD15 was up-regulated after LV108 treatment. Earlier studies found that the body weight and the lipid levels of rats in A group were significantly lower than M group. It is tempting to speculate that LV108 decreased the FFAs in the serum, which can accumulate in the liver, resulting in an up-regulation of ABHD15 expression. Collectively, ABHD15 might be an intriguing new target to research the effect of probiotics on NAFLD, as it impacts on adipogenesis and apoptosis.

Despite the predominant role of mitochondria, peroxisomes are also the key players in lipid metabolism.^50^ Peroxisomes might play a role in the early development of NAFLD and appear to be a potential target for treatment and prevention of NAFLD. PTS-2-containing PEX6 was reduced in the liver of db/db mice, which might indicate peroxisomal biosynthesis and functional maintenance.^51^ We found that compared with the M group, the expression level of PEX6 was up-regulated after LV108 treatment. It might indicate peroxisome biosynthesis was recovered to a relatively normal state and the liver lipid metabolism was restored to balance.

Phosphoglycerate kinase (PGK) and glyceraldehyde-3-phosphate dehydrogenas (GAPDH) are also involved in the metabolism. GAPDH is involved in step 1 of the sub-pathway for the synthesis of pyruvate from d-glyceraldehyde-3-phosphate. PGK is involved in step 2 of the sub-pathway for the synthesis of pyruvate from d-glyceraldehyde-3-phosphate. They are key enzymes in the glycolysis process. The lack of these two enzymes may cause disorders in the metabolic function of the organism.^52^ Compared with the model group, the expression levels of PGK and GAPDH were significantly up-regulated after LV108 treatment. In addition, KEGG pathway analysis shown that the phenylalanine, tyrosine and tryptophan biosynthesis also changed significantly. At the same time, differentially expressed proteins were also enriched in cysteine and methionine metabolism, biosynthesis of amino acids, and tyrosine metabolism. These indicate that LV108 has a regulatory effect on the metabolic function of the organism. It regulates metabolism-related proteins to restore metabolic balance, thereby alleviating NAFLD.

NAFLD is also related to the development and progression of liver cancer. In this study it was observed that compared with the N group, zinc-alpha2-glycoprotein (ZAG) and eukaryotic initiation factor 2B (elF2B) expression levels were up-regulated in M group, but could be corrected after LV108 treatment. ZAG, a lipid mobilizing factor, is a novel adipokine, which may be involved in the local regulation of adipose tissue function.^53^ ZAG is overexpressed in malignant tumors such that it may serve as a liver cancer marker.^54^ ZAG is actively involved in both inhibition of tumor growth and proliferation,^55^ inhibit the enzyme mediated tumor invasion and activate apoptosis.^56^ The high expression of ZAG in M group might indicate functional maintenance. eIF2B is a guanine nucleotide-exchange factor which mediates the exchange of GDP (bound to initiation factor eIF2) for GTP, thus regenerating the active.^57^ eIF2B plays a key role in the translation and regulation of mRNA. In cancer cells, eIF2B-mediated translational reprogramming protects cancer cells from apoptosis.^58,59^ LV108 restored the expression of ZAG and eIF2B, reducing the risk of continued development of NAFLD.

In conclusion, the present research first adopted the method of proteomics to carry out differential analysis of liver proteome on NAFLD model rats treated with probiotic. 26 important differentially expressed proteins were identified and the major functions were annotated as protein binding, cell part, and single-organism process. These proteins are mainly related to apoptosis and lipid metabolism. Bioinformatics and biological assay of these altered proteins will likely contribute to a better understanding of the molecular mechanisms of the effect of probiotics on NAFLD. However, the precise molecular mechanisms of action requires verification through further studies.

## Compliance with ethics guidelines

All institutional and national guidelines for the care and use of laboratory animals were followed.

## Conflicts of interest

Hengxian Qu, Hongbo Yu, Ruixia Gu, Dawei Chen, Xia Chen, Yingping Huang, Wenbo Xi and Yujun Huang declare that they have no conflict of interest.

## Abbreviations

NAFLD: Non-alcoholic fatty liver disease
TMT: Tandem mass tag
GO: Gene ontology
KEGG: Kyoto encyclopedia of genes and genome
PRM: Parallel reaction monitoring
BFAR: Bifunctional apoptosis regulator
Cyt-C: Cytochrome *c*

## Supplementary Material

RA-008-C8RA06771F-s001
